# Video-Assisted Thoracoscopic Surgery vs Thoracotomy for Non-Small Cell Lung Cancer Greater Than 5 cm: Is VATS a feasible approach for large tumors?

**DOI:** 10.1186/s13019-020-01305-w

**Published:** 2020-09-18

**Authors:** Güntuğ Batihan, Kenan Can Ceylan, Ozan Usluer, Şeyda Örs Kaya

**Affiliations:** Dr Suat Seren Chest Diseases and Surgery, Medical Practice and Research Center, Department of Thoracic Surgery, University of Health Sciences, Yenişehir, Gaziler Street 331, 35110 Izmir, Turkey

**Keywords:** Large tumors, Lung cancer, Thoracotomy, Video-assisted thoracoscopic surgery, This study presented in european respiratory congress 2019, Madrid as a poster discussion

## Abstract

**Background:**

VATS lobectomy is a recommended surgical approach for patients with early-stage lung cancer. However, it is still controversial in locally advance disease. This study was conducted to compare intraoperative and postoperative results of VATS and thoracotomy in patients with tumors greater than 5 cm.

**Methods:**

From January 2014 to December 2018, 849 patients underwent lobectomy or pneumonectomy for the treatment of non-small-cell lung cancer at our center. The inclusion criterion of this study was patients who underwent anatomic lung resection for lung cancer with tumors larger than 5 cm((≥ T3). The patients were divided into two groups: those who underwent video-assisted thoracoscopic surgery (*n* = 24) and those who underwent thoracotomy (*n* = 36). Patient characteristics, intraoperative and postoperative results were evaluated by review of the hospital records.

**Results:**

In the VATS group, mean drainage time and postoperative length of hospital stay were significantly shorter than the thoracotomy group. Kaplan–Meier survival curves showed that overall and recurrence-free survival was longer in the VATS group and this result was statistically significant.

**Conclusions:**

According to the results of this study, we emphasize that VATS is a feasible surgical procedure for tumors larger than 5 cm.

## Background

Anatomic lung resection is still the gold standard treatment approach in the management of early-stage lung cancer [1, 2].

Developing technology and adaptive surgical techniques have led the traditional surgical methods to evolve towards minimally invasive surgery. In the early 1990s, video-assisted thoracoscopic surgery (VATS) lobectomy described as a safe and feasible treatment option for early-stage non-small cell lung cancer (NSCLC) and over the years, with increasing experience, VATS has become a routine procedure in this group of patients [3, 4].

One of the biggest reasons for this rapid adaptation to the minimally invasive surgery in thoracic surgery is the positive results of VATS regarding postoperative pain and patient comfort [5, 6].

Promising surgical outcomes and improvements in modified surgical instruments have allowed the expansion of indications of VATS. Several studies have demonstrated that VATS can also be applied in patients with locally advanced lung cancer [5–7].

Although there are encouraging studies on the application of VATS in locally advanced disease, tumor size is still a challenging factor for many surgeons.

Difficulties in retraction and manipulation of the lung can cause inadequate exposure and result in life-threatening complications. Therefore, it is still controversial to perform of the VATS technique in patients with large tumors. In our department, with the increasing experience in parallel with the number of VATS lobectomies performed, we began to prefer VATS for patients with tumors larger than 5 cm in recent years and we have achieved positive results in terms of the applicability of VATS. Therefore, in this retrospective study we aimed to present these results and evaluate the safety, efficacy, and feasibility of video-assisted thoracoscopic lobectomy in patients with tumors greater than 5 cm compared with open lobectomy.

## Methods

### Patients selection

This retrospective study was approved by the Institutional Review Board. The data in this study were collected retrospectively from hospital records.

In total, 849 patients underwent anatomical pulmonary resection (lobectomy or pneumonectomy) for non-small cell lung cancer in our center from January 2014 to December 2018. The inclusion criteria are defined as patients who underwent anatomic lung resection for lung cancer with tumors larger than 5 cm (≥ T3). Tumor size was defined according to the maximum diameter in the pathological specimens.

We aimed to investigate the surgical difficulty caused by the tumor only due to its size. Therefore, patients who underwent neoadjuvant chemotherapy/radiotherapy, chest wall resection, tracheobronchoplasty, and angioplasty were excluded. A total of 60 patients who fitted the criteria were included in the study.

Thorax computed tomography (CT), positron emission tomography (PET)-CT, and bronchoscopy were performed for all patients before surgery. If mediastinal lymph node enlargement or high FDG uptake was detected on PET-CT, mediastinal lymph node sampling via endobronchial ultrasound with real-time guided transbronchial needle aspiration (EBUS-TBNA) or mediastinoscopy was performed first.

The patients were divided into two groups as VATS and thoracotomy. Patient characteristics, preoperative status, surgical procedures, perioperative course, pathological findings, and long-term prognoses were evaluated by review of the hospital records.

The postoperative complication was defined as any complication occurring within 30 days after surgery and prolonged air leakage was defined as air leak more than 7 days after surgery.

Recurrence-free survival was defined as the duration from the day of operation to the day of the detection of local or regional relapses of the tumor.

Postoperative mortality was defined as death occurring within 30 days post-surgery.

All patients had regular follow-up visits every three months with specially trained personnel in our department.

### Operative Technique

All patients were placed in the lateral decubitus position under general anesthesia with selective one-lung ventilation. We performed VATS resection using a two/three-port non-rib-spreading technique. A 3–4 cm utility incision was placed at the fifth or sixth intercostal space (ICS) in the midaxillary line. A 1.5 cm anterior/camera port was placed in the anterior axillary line (at the 7th or 8th ICS) and a 1.5 cm posterior port was placed in the posterior axillary line at the same level of ICS. Anterior and posterior ports were protected with a 10.5-mm trocar. After completion of the resection the specimen was extracted in a plastic bag.

In the thoracotomy group, pulmonary resections were performed through the muscle-sparing lateral thoracotomy using a 15 to 20 cm lateral skin incision. The fifth or sixth ICS was used. The major vascular branches were ligated and transfixed with non-absorbable sutures (mostly 2–0 or 1–0 silk). The pulmonary parenchyma, lobar and main bronchi were transected and closed with a surgical stapling device. We choose to cut open the bronchus when the tumor was close to the surgical margin.

### Statistical analysis

Statistical analysis was performed using SPSS 25.0 (SPSS Inc., Chicago, IL, USA).

Continuous variables, expressed as mean value ± standard deviation (SD), were compared by unpaired Student's t-tests; categorical variables were analyzed using Chi-square tests. DFS and OS were estimated using the Kaplan–Meier method. Cox regression analyzes were performed to define factors that would be effect disease-free and overall survival. Statistical significance was set at P-value < 0.05 (All P values presented were 2-sided).

## Results

From January 2014 to December 2018 a total of 60 patients with tumors greater than 5 cm who underwent lung resection were included the study. There were 57 men and 3 women. The mean age was 62.6 ± 8.2 years, with a range of 43 to 83. Among these patients, 24 of them were included in the VATS group and 36 of them were included in the thoracotomy group. Patients’ major characteristics were listed in Table 1.

**Table 1:** Patient characteristics

Variables	VATS (*n* = 24)	Thoracotomy (*n* = 36)	*P* Values
Mean age(years) (mean ± SD)	62.17 ± 1.61	63.19 ± 2.09	0.66
Sex (n (%))			
Male	22(91.7)	35(97.2)	0.33
Female	2(8.3)	1(2.8)	
**Co-morbidities (n (%)**			0.923
None	15(62.5)	19(52.8)	
Heart disease	1(4.2)	4(11.1)	
Hypertension	2(8.3)	3(8.3)	
Diabetes mellitus	2(8.3)	4(11.1)	
Chronic obstructive pulmonary disease	4(16.7)	6(16.7)	
Respiratory function test (mean ± SD)			
FEV1(%)	78 ± 16.3	73 ± 13.3	0.196
FEV1(lt)	2.34 ± 0.64	2.23 ± 0.58	0.344

*FEV* Forced expiratory volume, *SD* Standard deviation

There weren’t seen any significant differences in variables of mean age, gender, co-morbidities, and respiratory functions between thoracotomy and VATS groups (Table 1).

Intraoperative and postoperative complication rates were similar between VATS and thoracotomy groups (Table 2). Postoperative complications were summarized in Table 3.

**Table 2:** Perioperative and postoperative data

Variables	VATS (*n* = 24)	Thoracotomy (*n* = 36)	*P* Values
**Maximum dimension of the tumor (cm) (mean ± SD)**	6.93 ± 1.61	7.43 ± 2.09	0.26
**Pathology (n (%))**			0.031
Adenocarcinoma	14(58.3)	23(63.9)	
Squamous cell carcinoma	7(29.2)	11(30.6)	
Large cell carcinoma	3(12.5)	2(5.6)	
**Operation (n (%))**			0.237
Lobectomy	22(91.6)	26(72.2)	
Bilobectomy	1(4.2)	10(27.8)	
Pneumonectomy	1(4.2)	0(0)	
**Operative duration (minutes) (mean ± SD)**	228.75 ± 91.88	260.17 ± 102.78	0.784
**Overall number of nodal stations dissected (mean ± SD)**	5.13 ± 1.26	4.89 ± 1.03	0.352
**Intraoperative complication (n (%))**	1(5)^a^	2(5.6)	0.240
**Drainage time(days) (mean ± SD)**	5.00 ± 3.43	8.14 ± 4.95	0.009
**Length of stay (days) (mean ± SD)**	5.42 ± 2.32	9.11 ± 5.31	0.001
**Nodal status (n (%)**			0.494
**N0**	17(70.8)	22(61.1)	
**N1**	5(20.8)	7(19.4)	
**N2**	2(8.3)	7(19.4)	
**Postoperative pathological staging (n (%))**			0.914
Stage II	9(37.5)	14(38.9)	
Stage III	15(62.5)	22(61.1)	
**Follow-up time(months) (mean ± SD)**	27.67 ± 6.02	26.36 ± 5.24	0.784
**Adjuvant therapy (n (%))**	17(70.8)	26(72.2)	1.00
**Time interval between surgery and adjuvant therapy (days) (mean ± SD)**	43.71 ± 14.45	49.96 ± 15.04	0.081

*SD* Standard deviation

^a^A total of 25 patients started with VATS, one patient converted into thoracotomy because of intraoperative bleeding

**Table 3:** Postoperative complications

Postoperative Complications (n (%))	VATS (*n* = 24)	Thoracotomy(*n* = 36)	*P* values
Prolonged air leakage	4(16.7)	9(25)	
Hemorrhage	1(4.1)	1(2.8)	
Pneumonia	3(12.5)	2(5.6)	
Empyema	0(0)	1(2.8)	
Bronchopleural fistula	0(0)	1(2.8)	
**Total**	**8(33.3)**	**14(38.9)**	**0.734**

A total of 25 patients started with VATS, one (%4) patient converted into thoracotomy because of intraoperative bleeding from the pulmonary artery. In this case, the laceration in the pulmonary artery was successfully repaired after conversion to thoracotomy.

In the VATS group, mean drainage time and postoperative length of hospital stay were significantly shorter than the thoracotomy group (*p* = 0.009; *p* = 0.001).

The proportion of patients receiving adjuvant therapy was similar in the VATS and thoracotomy groups but the time interval between surgery and adjuvant therapy was shorter in the VATS group than thoracotomy, but statistical significance was in borderline (*p *= 0.081).

**Fig. 1:**
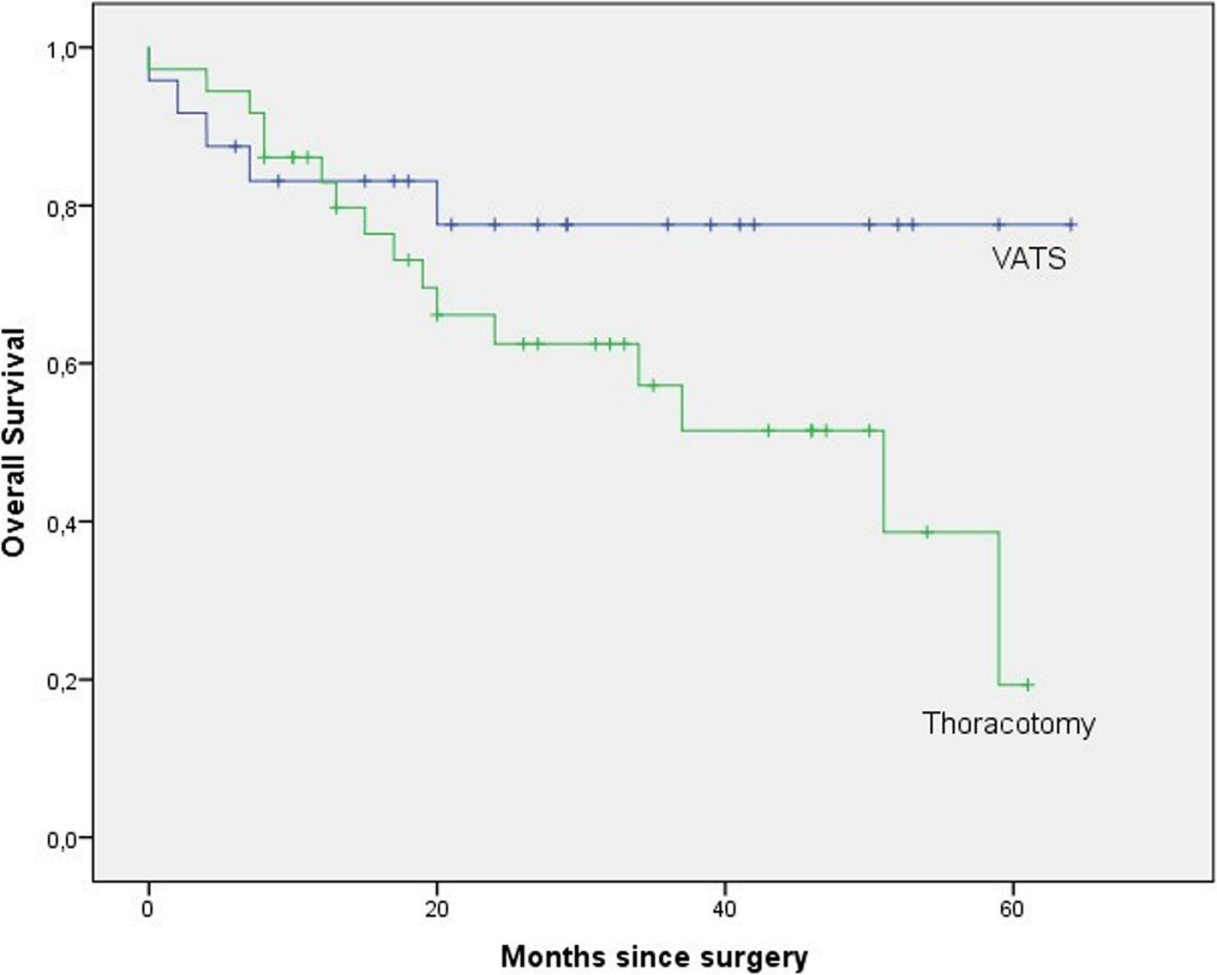
Overall survival (OS) of patients with lung cancers of > 5 cm after resection (VATS: video-assisted thoracoscopic surgery)

**Fig. 2:**
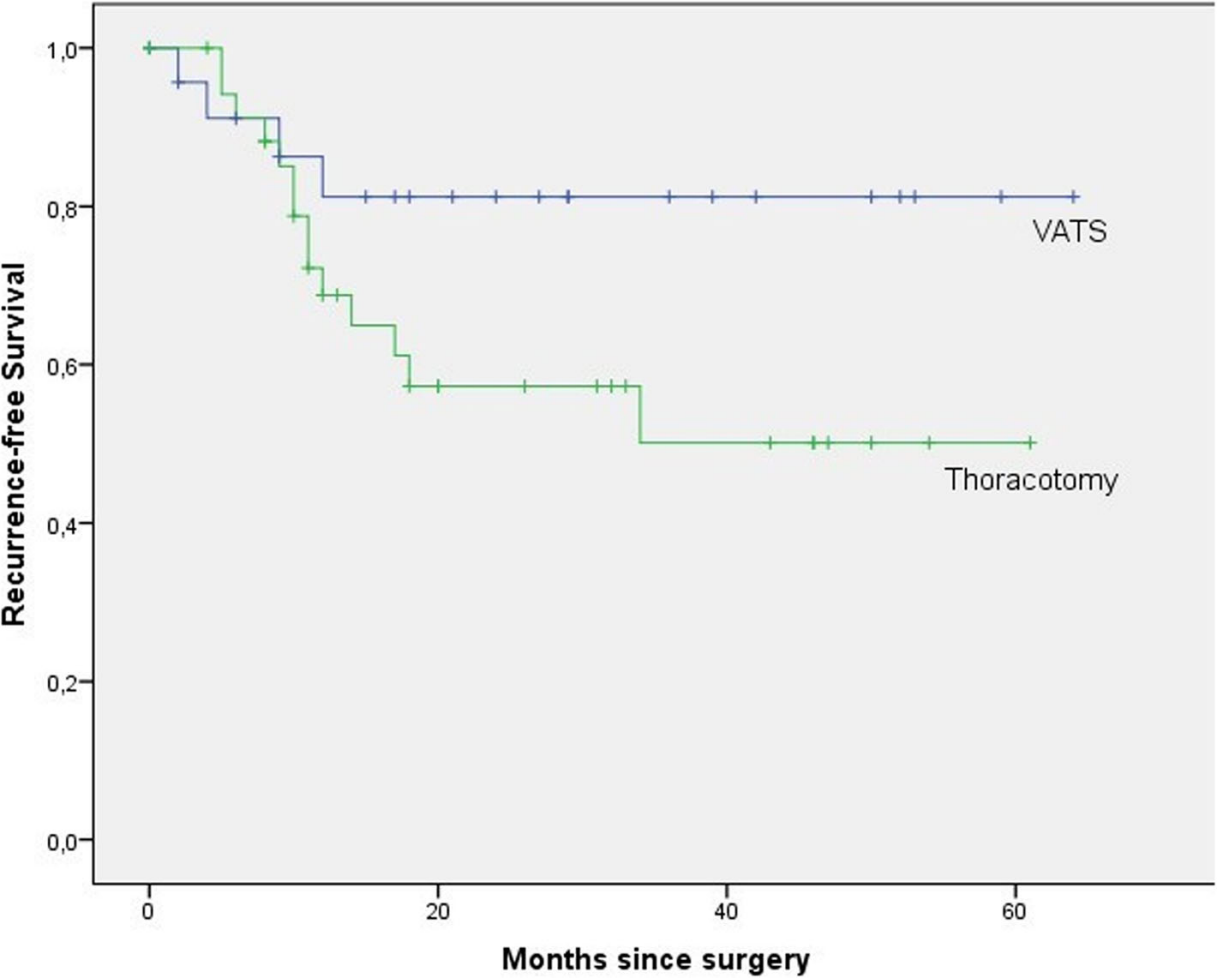
Recurrence-free survival of patients with lung cancers of > 5 cm after resection (VATS: video-assisted thoracoscopic surgery)

We also compared overall and recurrence-free survival between VATS and thoracotomy groups (Table 4) and interestingly, Kaplan–Meier survival curves revealed that overall and recurrence-free survival was longer in VATS group and this result was statistically significant (*p *= 0.056/0.031) (Fig. 1 and 2).

**Table 4:** Survival and recurrence

Variables	VATS (*n *= 24)	Thoracotomy (*n* = 36)	*P* Values
**Recurrence free survival(months)**	53.29	37.65	0.031
**Recurrence (%)**			
1-year	81.2	78.8	
3-year	81.2	50.2	
**Overall survival (months)**	51.31	38.49	0.056
**Survival (%)**			
1-year	77.6	82.9	
3-year	77.6	57.2

According to the multivariable cox regression analysis, disease-free interval and pathological subtypes were independent predictors of overall survival (*p *= 0.002; *p *= 0.044) (Table 5). The same analyses also revealed that the preferred surgical approach (VATS or thoracotomy) and pathological stage were independent predictors of disease-free survival (*p =* 0.023; *p *= 0.003) (Table 6).

**Table 5:** Results of multivariate Cox regression model of overall survival

Covariates	Coefficient	Standard error	*P*-value	Hazard ratio	95% Cl
Age	*0.036*	0.049	0.457	1.037	0.942–1.141
Gender (0: Male, 1: Female)	-12.569	695.002	0.986	0.000	0.000
FEV1	-3.873	2.953	0.190	0.021	0.000–6.781
Tumor size	-0.259	0.205	0.205	0.771	0.516–1.153
Approach (0: VATS, 1: Thoracotomy)	0.496	0.936	0.064	1.609	0.978–3.812
Stage (0: II, 1: III)	-0.562	1.070	0.599	0.570	0.070–4.638
Disease free interval	*-0.165*	0.052	0.002	0.848	0.765–0.939
Pathological subtype			0.044		
Squamous cell ca	Ref				
Adeno ca	-2.655	1.157	0.022	0.070	0.007–0.679
Large cell ca	-2.099	1.532	0.171	0.123	0.006–2.469
Comorbidity			0.409		
Coronary artery disease	1.141	1.582	0.470	3.132	0.141–69.567
Diabetes mellitus	2.889	1.734	0.096	17.977	0.601–537.679
COPD	-2.106	1.899	0.267	0.122	0.003–5.034
Hypertension	-12.212	850.563	0.989	0.00	0.000-
Time interval between surgery and adjuvant therapy	0.058	0.034	0.049	1.059	0.991–1.132

*FEV* Forced expiratory volume, *COPD* Chronic obstructive pulmonary disease, *Cl* Confidence Interval, *Ref* Reference variable

**Table 6:** Results of multivariate Cox regression model of disease free survival

Covariates	Coefficient	Standard error	*P*-value	Hazard ratio	95% Cl
Age	*0.015*	0.045	0.737	1.015	0.930–1.108
Gender (0: Male, 1: Female)	-13.419	1112.087	0.990	0.000	0.000
Tumor size	-0.731	0.353	0.380	0.481	0.241–1.961
Approach (0: VATS, 1: Thoracotomy)	1.989	0.878	0.023	7.311	1.309–40.827
Stage (0: II, 1: III)	4.886	1.657	0.003	132.403	5.141–3405.902
Pathological subtype			0.156		
Squamous cell ca	Ref				
Adeno ca	0.268	0.679	0.693	1.308	0.346–4.950
Large cell ca	2.583	0.679	0.054	13.234	0.959–182.552
Time interval between surgery and adjuvant therapy	0.009	0.023	0.681	1.009	0.965–1.055
Overall number of nodal stations dissected	-0.008	0.405	0.985	0.993	0.449–2.195
Nodal status			0.075		
N0	Ref				
N1	-3.591	1.579	0.023	0.028	0.001–0.609
N2	-16.790	596.799	0.978	0.000	0.000

## Discussion

Multiple large-scale clinical studies have shown that VATS lobectomy has an apparent advantage over the thoracotomy in terms of length of hospital stay, drainage time, postoperative pain, and patient comfort [8–10]. Due to its proven superiority, VATS lobectomy has been recommended by the National Comprehensive Cancer Network (NCCN) guidelines as the preferred approach for early-stage NSCLC [11]. Besides these encouraging data, there are still doubts about the use of VATS in locally advanced lung cancer.

Liang et al. analyzed 133 patients with a tumor larger than 5 cm and compared VATS with open lobectomy. In this study, the VATS group had shorter operative duration and less intraoperative bleeding but there wasn't seen any significant difference in terms of mean drainage time, postoperative length of stay, and survival [12]. Nakano et al. retrospectively analyzed 68 patients who underwent anatomical pulmonary resection for primary lung cancer of > 5-cm diameter and reported significantly less intraoperative bleeding and shorter length of postoperative hospital stay in the VATS group. They also reported similar recurrence-free and overall survival between two groups [13].

In our study, there weren’t seen any significant differences in postoperative complications between VATS and thoracotomy groups. The most common postoperative complication in both groups was prolonged air leakage. However, most of the patients whose complication was categorized as prolonged air leakage had a pleural space problem instead of a massive air leakage.

To reduce the risk of prolonged air leakage, we preferred to apply the fissureless-VATS technique. In this technique, the entire dissection of the interlobar fissure is left to the end and performed by staplers instead of dividing the overlying parenchyma to expose the pulmonary artery [14–16].

Similar to the previous studies, we detected shorter drainage time and postoperative hospital stays in the VATS group [8–10]. But differently in our study, patients in the VATS group had significantly longer overall and recurrent-free survival. There are some hypotheses about this issue in the literature [17, 18]:

1- It was shown that VATS associated with less surgical injury and this situation can cause less immune suppression.

2- Rapid recovery after the operation allows patients to undergo adjuvant therapy sooner. In our study, we detected a shorter interval between surgery and adjuvant therapy in the VATS group, but statistical significance was in borderline (*p *= 0.081).

3- The shorter length of hospital stay will also reduce exposure to nosocomial infections (More likely to be associated with postoperative mortality).

Common inference from our own experience and few similar studies is the major concern about applying VATS for tumors larger than 5 cm is the problem of the retraction and manipulation of the lung. Difficulty in providing a good view may cause unnecessary prolongation of the operation time and life-treating complications.

The Port placements and cooperation with an assistant are very important to provide sufficient exposure. The presence of an experienced anesthesia team is vital to maintain successfully selective one-lung ventilation. Another important point is to decide to convert open surgery with the right timing. Emergency conversions significantly increase the risk of mortality and morbidity [19]. An experienced surgeon should be able to decide to convert thoracotomy by predicting the possible complications. In our cases, we didn't refrain from opening the third port if needed and in 1 case converted to thoracotomy because of vascular injury.

Another important point in patients with large tumors that difficulty in lymph node dissection.

Systematic lymph node resection is an important part of surgical treatment for NSCLC and it is associated with long-term survival. There were concerns about the adequacy of VATS in lymph node dissection in the early days of thoracoscopic surgery, but many studies have shown that thoracoscopic systematic lymphadenectomy was as effective as that performed via thoracotomy [20–22]. We performed complete mediastinal lymph node dissection in all cases, and there were no significant differences between group V and group T in the number of dissected lymph node stations (*p* = 0.352).

Sometimes it can be difficult to remove the specimen from the thoracic cavity in the VATS procedure. This difficulty occurs especially in the presence of a large tumor and narrow intercostal space and sometimes results in rupture of the endoscopic bag.

As a solution to this unwanted situation, in patients with a large tumor and narrow intercostal space, we used double bags while removing resection material from the thoracic cavity. Extending the incision can also help, but often the main problem is the narrowness of the intercostal space rather than the size of the incision.

Although we try to avoid possible bias with the strict inclusion criteria, bias is inevitable due to its retrospective nature. With increasing experience, our preference for VATS has increased over time in patients with large tumors therefore, in the present study patient selection criteria were not defined clearly. The survival advantage detected in the VATS group should be confirmed by prospective studies with larger patient groups. The number of patients in the study is low due to being a subgroup study. Especially the advantage of survival in the VATS group should be confirmed by multi-center studies.

## Conclusion

In conclusion, this current study demonstrates significant differences in drainage time, length of hospital stays, overall and recurrence-free survival in favor of VATS. Therefore, according to these results, we emphasize that VATS is a feasible surgical procedure for tumors larger than 5 cm. Difficulties may be experienced in the retraction of the lung and providing adequate exposure for safe dissection but this kind of issue can be overcome with the proper placement of ports, the use of appropriate surgical instruments, and teamwork.

## Data Availability

Not applicable.
